# Effect of ovariectomy on the progression of chronic kidney disease-mineral bone disorder (CKD-MBD) in female Cy/+ rats

**DOI:** 10.1038/s41598-019-44415-9

**Published:** 2019-05-28

**Authors:** Colby J. Vorland, Pamela J. Lachcik, Elizabeth A. Swallow, Corinne E. Metzger, Matthew R. Allen, Neal X. Chen, Sharon M. Moe, Kathleen M. Hill Gallant

**Affiliations:** 1 Department of Nutrition Science, Purdue University, West Lafayette, IN 47907 USA; 2 Department of Anatomy and Cell Biology, Indiana University School of Medicine, Indianapolis, 46202 IN USA; 3 Division of Nephrology, Department of Medicine, Indiana University School of Medicine, Indianapolis, 46202 IN USA; 4 Roudebush Veterans Affairs Medical Center, Indianapolis, IN 46202 USA

**Keywords:** Endocrine system and metabolic diseases, Kidney

## Abstract

Male Cy/+ rats have shown a relatively consistent pattern of progressive kidney disease development that displays multiple key features of late stage chronic kidney disease-mineral bone disorder (CKD-MBD), specifically the development of cortical bone porosity. However, progression of disease in female Cy/+ rats, assessed in limited studies, is more heterogeneous and to date has failed to show development of the CKD-MBD phenotype, thus limiting their use as a practical model of progressive CKD-MBD. Animal and human studies suggest that estrogen may be protective against kidney disease in addition to its established protective effect on bone. Therefore, in this study, we aimed to determine the effect of ovariectomy (OVX) on the biochemical and skeletal manifestations of CKD-MBD in Cy/+ female rats. We hypothesized that OVX would accelerate development of the biochemical and skeletal features of CKD-MBD in female Cy/+ rats, similar to those seen in male Cy/+ rats. Female Cy/+ rats underwent OVX (n = 8) or Sham (n = 8) surgery at 15 weeks of age. Blood was collected every 5 weeks post-surgery until 35 weeks of age, when the rats underwent a 4-day metabolic balance, and the tibia and final blood were collected at the time of sacrifice. OVX produced the expected changes in trabecular and cortical parameters consistent with post-menopausal disease, and negative phosphorus balance compared with Sham. However, indicators of CKD-MBD were similar between OVX and Sham (similar kidney weight, plasma blood urea nitrogen, creatinine, creatinine clearance, phosphorus, calcium, parathyroid hormone, and no cortical porosity). Contrary to our hypothesis, OVX did not produce evidence of development of the CKD-MBD phenotype in female Cy/+ rats.

## Introduction

Chronic kidney disease (CKD) affects approximately 13.4% of adults worldwide^1^, and prevalence and progression of the disease differ based on biological sex. Estimation of the global prevalence of CKD is higher in women than men^1^, but a large cohort study showed a higher proportion of males than females at end stage kidney disease^2^. A meta-analysis of 68 studies on nondiabetic kidney disease concluded that kidney function declines more slowly in women than men^3^. Population studies show that end-stage kidney disease incidence is higher in men than premenopausal women, but sex differences begin to lessen around menopausal years^4^. In concordance, ovariectomy (OVX) has been demonstrated to accelerate kidney disease in various animal models^5–10^, while exogenous estradiol administration attenuates the disease^6–13^. Similarly, in premenopausal women, bilateral oophorectomy at age ≤45 years was associated with an elevated risk of CKD as assessed by estimated glomerular filtration rate^14^. However, a meta-analysis of hormone replacement studies in postmenopausal women found no significant effect on albuminuria or proteinuria when assessed together but in a subgroup analysis of studies assessing only albuminuria there was a small favorable effect of hormone replacement in lowering albuminuria^15^. Further, some divergent studies have found no effect^16,17^ or even a protective effect^18,19^ of estrogen loss on kidney disease progression. In addition to the impact on kidney disease progression, estrogen has well-established protective effects on bone^20^. This is highly relevant as a common co-morbidity of CKD is mineral bone disorder (CKD-MBD). CKD-MBD is characterized by biochemical abnormalities of mineral metabolism, bone disease, and vascular or other soft tissue calcification that result in an increased risk for cardiovascular events, bone fractures, and death^21^. Estrogen may modulate the main biochemical indicators of CKD-MBD, fibroblast growth factor-23 (FGF-23), parathyroid hormone (PTH), and 1,25-dihydroxyvitamin D (1,25D), either directly or indirectly^22^. Thus, estrogen appears to be protective against kidney failure and associated co-morbid conditions. However, biological sex differences in the manifestation and progression of CKD-MBD through the stages of CKD are understudied.

The Cy/+ rat model of CKD is unique in that it has been characterized as a spontaneous slowly progressive model of CKD that exhibits all the key features of CKD-MBD and can be studied at earlier to later stages of disease progression^23,24^. The phenotype is the result of a missense mutation in *Anks6* that encodes for SamCystin, and results in renal cyst formation^25^. Male Cy/+ rats experience a clear elevation in blood urea nitrogen (BUN) by 10 weeks of age^23^ and show all features of late stage CKD-MBD by 35 to 38 weeks of age, including changes in additional plasma biochemistries such as creatinine, hematocrit, phosphorus and calcium, regulatory hormones PTH and FGF23, bone abnormalities (particularly cortical porosity), and vascular calcification^24,26,27^. On the contrary, female Cy/+ rats do not experience an elevation in BUN until around 40–44 weeks of age and even then are not comparable to levels in 10-week-old males^28,29^. This has resulted in minimal use of female Cy/+ rats in studies on CKD-MBD. Therefore, the aim of this study was to determine the effect of OVX on the biochemical and skeletal manifestations of CKD-MBD in Cy/+ female rats. We hypothesized that OVX would accelerate development of the biochemical and skeletal features of CKD-MBD in female Cy/+ rats, similar to those seen in male Cy/+ rats, by 35 weeks of age. Because most women with CKD are postmenopausal or amenorrhoeic due to the disease^30^, this model would be translationally relevant to a large percentage of women with CKD who have concurrent postmenopausal osteoporosis.

## Materials and Methods

### Animals

Sixteen female Cy/+ rats were studied from the Cy rat colony maintained at Purdue University, which was rederived from the Indiana University School of Medicine colony maintained by Dr. Sharon Moe. Heterozygosity for the Anks6 mutation was determined by ear punch and genotyping (Transnetyx, Memphis, TN). Rats were randomly assigned to shoe-box cages (2 rats per cage), and within each cage, randomly assigned to undergo OVX (N = 8) or Sham (N = 8) surgery at 15 weeks of age (described below). Blood was drawn at 10, 20, 25, 30, and 35 weeks of age through the jugular vein after rats were anesthetized with isoflurane. Rats were fed standard rat chow containing 0.7% phosphorus and 1.0% Ca (Envigo Teklad 2018, Madison, WI) and water *ad libitum* until 24 weeks of age, at which time they were switched to an *ad libitum* casein-based diet (0.7% phosphorus and 0.7% calcium) which we have previously shown to lead to more consistent and accelerated kidney decline in Cy/+ males (TD.04539, Envigo Teklad, Madison, WI)^24^. Rats were fed this diet until sacrifice at 35 weeks of age. At 13 days prior to sacrifice, rats were transferred to wire-bottom metabolic cages and a four-day phosphorus and calcium balance was performed from 9 to 5 days prior to sacrifice. Five days prior to sacrifice, rats were transferred back to shoe-box cages. Body weights were taken weekly. The light-dark cycle was maintained from 6:30AM-6:30PM. Room temperature was held at ~21 °C and humidity ~27%. Experiments were performed in accordance with relevant guidelines and regulations, and this protocol was approved by the Purdue University Animal Care and Use Committee (protocol #1702001543).

### OVX and sham procedures

All appropriate steps were taken for performing aseptic surgery for OVX and Sham procedures. OVX procedures followed similarly to those described in the Harlan protocol (HUS-QREC-PRD-932, Issue 1, Revision 03). Briefly, rats were shaved on the dorsal midline where one incision of ~2 cm was made. The incision was pulled to the left side and a small ~10 mm incision was made through the abdominal wall. The ovary was externalized, and a silk ligature was tied between the end of the uterine horn and the ovary and ovarian artery. The ovary was excised and removed. The abdominal wall was closed with an absorbable suture and then the skin was pulled to the opposite side to remove the other ovary by the same method. Sham-operated rats underwent the same surgical procedure, excluding the ligation and removal of the ovaries.

### Tissue collection

At sacrifice, rats were anesthetized with isoflurane, the thoracic cavity was opened, and blood was collected from the vena cava resulting in death by exsanguination. Kidneys and uteri were excised and weighed. The left tibia was excised, cleaned of surrounding soft-tissue, and stored in 10% neutral buffered formalin for 3 days, then transfer to 70% ethanol and stored at −20 °C until the time of microcomputed tomography (µCT) analysis^31^.

### Phosphorus and calcium balance and percent net absorption

Over the four days of metabolic balance, all urine and feces were collected, and diet was weighed daily to assess 4-day average phosphorus and calcium balance and net absorption. Feces and diet were ashed in a muffle furnace (Thermolyne Sybron Type 30400, Dubuque, IA) for 10 days at 600 °C. Feces were then diluted 1400X and diet 60X with 2% nitric acid. Urine was diluted 11X with 2% nitric acid. Phosphorus and calcium in urine, feces, and diet were quantified by inductively coupled plasma-optical emission spectrophotometry (ICP-OES; Optima 4300DV, Perkin Elmer, Shelton, CT). Urine creatinine was determined by colorimetric method (QuantiChrom Creatinine Assay Kit; BioAssay Systems, Hayward, CA). Four-day phosphorus balance was calculated as dietary phosphorus intake (mg/d) minus urine and fecal phosphorus excretion (mg/d), and percent net phosphorus absorption as phosphorus intake (mg/d) minus fecal excretion (mg/d)/phosphorus intake (mg/d). Calcium balance and percent net calcium absorption were calculated similarly.

### Plasma biochemistries

Plasma stored at −80C was thawed and analyzed for mineral biochemistries. Phosphorus, calcium, BUN, and creatinine were measured by colorimetric assay (Phosphorus Kit: Pointe Scientific Inc., Canton, MI; Calcium Kit: Pointe Scientific Inc., Canton, MI; BUN: QuantiChrom Urea Assay Kit, BioAssay Systems, Hayward, CA; Creatinine: QuantiChrom Creatinine Assay Kit; BioAssay Systems, Hayward, CA). Estradiol and intact PTH (iPTH) were measured by enzyme-linked immunosorbent assay (Calbiotech Inc., El Cajon, CA; Alpco, Salem, NH, respectively).

### Microcomputed tomography

Proximal tibiae were analyzed by µCT (Skyscan 1172, 12 µm resolution) using protocols similar to our previous studies^27^. Trabecular microarchitecture was obtained from a 1 mm region of interest selected approximately 1 mm distal to the tibial growth plate. Bone parameters assessed included trabecular bone volume/tissue volume (BV/TV, %), trabecular thickness (Tb.Th), trabecular number (Tb.N), and trabecular separation (Tb.Sp). Cortical bone analysis was performed on a single slice located 1.5 mm distal from the metaphysis region of analysis with outcome parameters including cortical bone area (Ct.Ar), cortical thickness (Ct.Th), and cortical porosity.

### Statistics

Group differences for plasma biochemistries with multiple timepoints (calcium, phosphate, creatinine, BUN, and estradiol) were compared with a linear mixed model with rat ID as a random effect, with post hoc pairwise comparisons. Unpaired, two-tailed t-tests were performed for comparison of group differences in endpoint iPTH, uterine and kidney weights, mineral balance and net absorption, and bone outcomes between OVX and Sham. Estradiol measures were log-transformed because they were not normally distributed, and the reported p values reflect statistical comparisons of the transformed values. Because of unequal variances for uterine weight and all bone outcomes, the Satterthwaite test was used to test for differences. Because two rats were housed per cage, all statistical comparisons were repeated with a linear mixed model with cage ID as a random effect. The cage effect was small and did not alter conclusions with the exception of BUN, for which results are reported as the model including cage as a random effect. All other results were reported without cage ID as a random effect. Statistical significance was set at α < 0.05. All statistical tests included all n = 16 rats. Statistical Analysis Software version 9.4 (SAS Institute, Cary, NC) was used for all statistical analyses. Results are reported as mean ± SEM.

## Results

**Figure 1:**
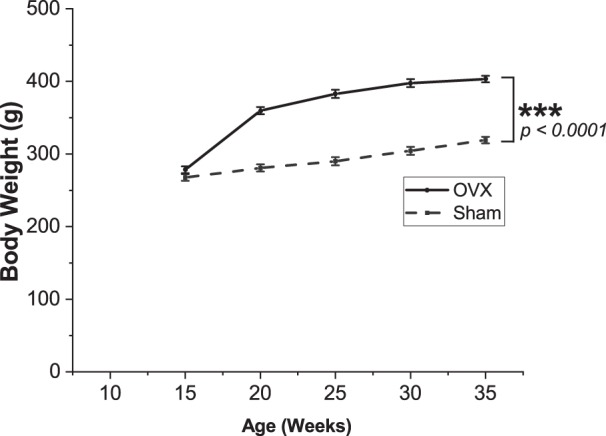
OVX resulted in higher body mass relative to Sham. Values presented are mean ± SEM. ***p < 0.0001 between groups.

**Figure 2:**
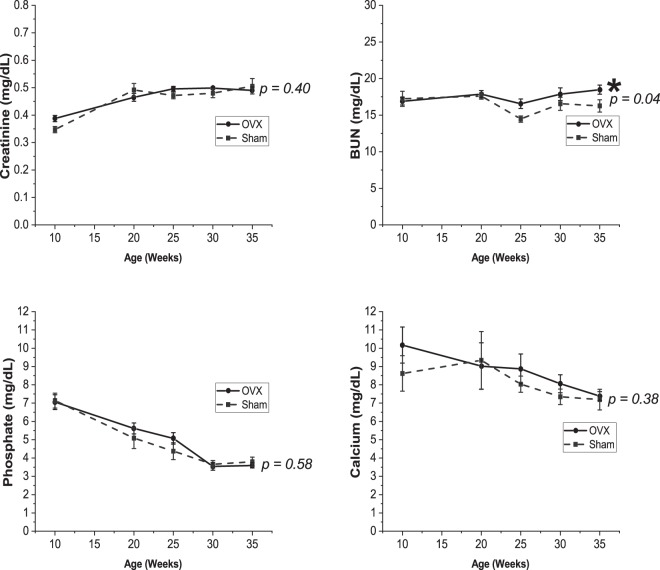
Plasma biochemistries over time between OVX and Sham surgery. Plasma creatinine, phosphate, and calcium were not different between the groups. BUN was statistically higher in the OVX group. Values presented are mean ± SEM. For comparison reference range for Cy/+ male rats at 34–35 weeks based on Moe *et al.* in^24,32^: BUN (~60–107 mg/dL), phosphate (~6.8–11.6 mg/dL), calcium (~8.5–9.8 mg/dL). We have also measured plasma creatinine at 0.92 ± 0.06 mg/dL in 30 week Cy/+ males (*unpublished*).

OVX was deemed successful as evidenced by the lower estradiol at 35 weeks (1.5 ± 0.1 pg/mL vs 6.5 ± 2.4 pg/mL, p = 0.06 for group*time interaction, p = 0.01 for 35 week pairwise comparison between OVX and Sham, Table 1), as well as significantly lower uterine weight observed at 35 weeks (0.16 ± 0.01 g vs 0.78 ± 0.06 g, respectively; p < 0.0001), and greater increase in body weight after OVX compared with Sham (p < 0.0001, Fig. 1). Plasma creatinine (p = 0.40), phosphorus (p = 0.58), and calcium (p = 0.38) were not different between OVX and Sham groups. BUN was statistically higher in OVX compared with Sham, but with only a small difference in means at 35 weeks (18.5 ± 0.6 mg/dL vs 16.3 ± 0.8 mg/dL, p = 0.04) (Fig. 2), which has low practical relevance when compared to the values of ~60–107 mg/dL observed in our previous studies in Cy/+ male rats of similar age^24,32^. Creatinine clearance was not different between groups (4.2 ± 0.2 mL/min vs 4.1 ± 0.2 mL/min for OVX and Sham respectively, p = 0.83). In addition, kidney weight was not different between groups (1.417 ± 0.075 g vs 1.382 ± 0.050 g, p = 0.70).

**Table 1:** Estradiol at baseline (10 weeks, pre-surgery) and 35 weeks.

	OVX (n = 8)	Sham (n = 8)	*P*
Baseline estradiol (pg/mL)	4.0 ± 0.6	4.1 ± 1.1	0.99
35 week estradiol (pg/mL)	1.5* ± 0.1	6.5 ± 2.4	0.01

Values are mean ± SEM. *Less than the analytic sensitivity of 3 pg/mL.

**Figure 3:**
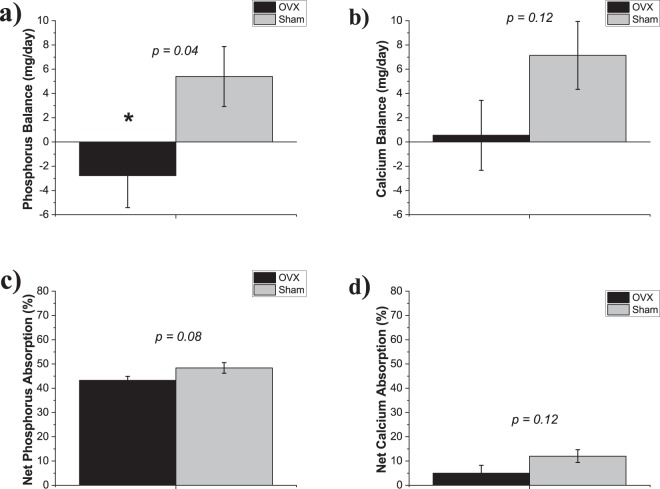
Mineral balance and net mineral absorption at 35 weeks by OVX or Sham surgery. (**a**) Phosphorus balance was lower in OVX rats vs Sham. (**b**) Calcium balance was not different between the groups. (**c**) Percent net phosphorus absorption and (**d**) percent net calcium absorption were not different between groups. Values presented are mean ± SE. *p = 0.04.

Phosphorus balance was lower in OVX rats compared to Sham (−2.8 ± 2.6 mg/day vs 5.4 ± 2.5 mg/day, p = 0.04), while calcium balance was also numerically lower but did not reach statistical significance (0.6 ± 2.9 mg/day vs 7.1 ± 2.8 mg/day, p = 0.12) (Fig. 3). Similarly, both percent net phosphorus and calcium absorption were numerically lower in OVX rats vs Sham, but neither reached statistical significance (43 ± 2% vs 48 ± 2%, p = 0.08; 5 ± 4% vs 12 ± 3%, p = 0.12, phosphorus and calcium, respectively) (Fig. 3).

**Figure 4:**
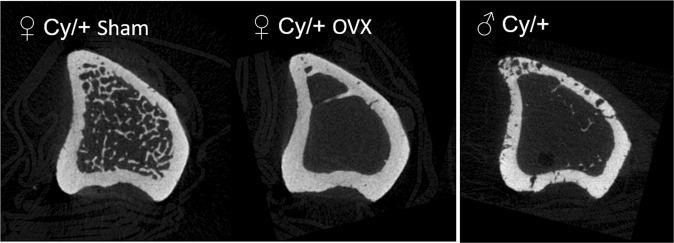
Micro-CT images of the proximal tibia. Representative crosssectional micro-CT images of the proximal tibia. From left to right: female Cy/+ Sham rats, female Cy/+ OVX rats (present study, Table 2), and for visual comparison, a representative image from a 35-week-old male Cy/+ rat showing prominent cortical porosity (*unpublished*) that is absent from both Sham and OVX female Cy/+ rats in the present study.

In the tibia, BV/TV and Tb.N were lower in OVX rats (p < 0.0001 for both), and Tb.Sp, Ct.Ar, and Ct.Th higher (p < 0.0001) compared with Sham (Table 2). Tb.Th was not different between groups (p = 0.17) (Table 2). No cortical porosity was noted for any rats (Sham nor OVX) (Fig. 4). Accordingly, no difference in iPTH at 35 weeks between groups was observed (138.7 ± 13.2 pg/mL vs 141.8 ± 20.1 pg/mL for OVX vs Sham, p = 0.90).

**Table 2:** Microstructural parameters of cancellous and cortical bone of the tibia measured by micro-CT.

	OVX (n = 8)	Sham (n = 8)	*P*
BV/TV (%)	0.99 ± 0.34	17.45 ± 1.59	<0.0001
Tb.N (1/mm^−1^)	0.14 ± 0.04	2.16 ± 0.13	<0.0001
Tb.Th (mm)	0.07 ± 0.008	0.08 ± 0.003	0.17
Tb.Sp (mm)	0.84 ± 0.02	0.27 ± 0.01	<0.0001
Ct.Ar (mm^2^)	5.93 ± 0.07	5.22 ± 0.07	<0.0001
Ct.Th (mm)	0.48 ± 0.004	0.39 ± 0.005	<0.0001

Values are mean ± SE. BV/TV (bone volume (BV)/Tissue volume (TV)); Tb.N (trabecular number); Tb.Th (trabecular thickness); Tb.Sp (trabecular separation); Ct.Ar (cortical bone area); Ct.Th (cortical thickness).

## Discussion

OVX of Cy/+ female rats produced the changes expected with OVX consistent with the postmenopausal condition that included higher body weight, lower uterine weight, and lower trabecular bone volume compared to Sham rats^33,34^. However, there was no indication that OVX advanced the CKD-MBD phenotype as evidenced by similar creatinine clearance, plasma creatinine, BUN, kidney weight, phosphorus, and calcium compared with Sham. And, although OVX produced expected changes in cancellous bone and cortical area and thickness, the increased cortical porosity that is the hallmark of the CKD-MBD phenotype in male Cy/+ rats^32^ was not observed in any rats. These findings were contrary to our hypothesis that OVX would promote the development of CKD-MBD in female Cy/+ rats to an extent where they could be a practical female model of CKD-MBD.

Sex differences in the progression of kidney disease have been observed in both rodents and humans, with some conflicting results. Some studies have shown an increase in disease progression in response to OVX. This has been demonstrated in sclerosis-prone ROP Os/+ mice^5^, 5/6 nephrectomized Wistar rats^6^, in rats with streptozotocin-induced diabetic nephropathy^7^, Dahl salt-sensitive rats^8^, and in female Imai rats that develop spontaneous hypercholesterolemia^10^. Further, administration of estradiol tended to mitigate the changes^6–8^. In female Imai rats, a protective or aggravative effect of estradiol on glomerulosclerosis was dependent on dose^10^ which may be mediated by growth hormone^35^. Additionally, exogenous estradiol in spontaneously hypertensive rats that underwent uninephrectomy, or in female db/db mice, male Imai rats, or male Sprague-Dawley rats has been demonstrated to reduce age-related kidney disease progression^9,11–13^. The estradiol metabolite 2-hydroxyestradiol has also been shown to be renoprotective in puromycin-aminonucleoside model of nephropathy^36^. Protective effects of estrogen on renal function have been suggested to be mediated by a reduction in extracellular matrix protein accumulation^37,38^.

In contrast to the above-mentioned studies, OVX did not worsen kidney disease in female Munich-Wistar rats that progressively develop glomerular injury with age nor 5/6 nephrectomized Wistar rats^16,17^. Further, some studies have even shown apparent benefits of OVX to renal outcomes: OVX in spontaneous hypertensive stroke-prone female rats increased survival and reduced renal vascular pathology compared to Sham surgery, which was reversed with estradiol administration^18^. Similarly, in context of hyperlipidemia in both analbuminemic and Zucker rats, OVX was protective against glomerulosclerosis, while exogenous estradiol worsened it^19,39^.

Previous studies in the Cy/+ rat have demonstrated that females do not develop the pronounced azotemia and fibrosis from cystic disease until much older ages compared with males^28,29^. A previous study of OVX was conducted in weanling Cy/+ female rats by Cowley *et al*.^40^. These rats underwent OVX at 4 weeks of age and did not exhibit subsequent changes in kidney weight, volume density of renal cysts, or BUN by 10 weeks of age compared to Sham rats. However, testosterone administration induced cystic disease progression^40^. Our findings confirm that this lack of effect of OVX on kidney disease progression is not limited to growing rats in the Cy/+ model of CKD. However, at odds with our results and those of Cowley *et al*. is a study by Stringer *et al*.^41^ in which Cy/+ rats underwent OVX at 6 weeks of age, and kidney function was evaluated at 12 weeks of age. These investigators observed that OVX of female rats hastened the progression of polycystic kidney disease, and likewise, orchidectomy in male Cy/+ rats slowed disease progression. The stark difference in ages studied, and particularly age of OVX, makes comparisons among these studies challenging. Our study models more closely a postmenopausal loss of ovarian function versus these prior studies in growing Cy/+ rats that model a deprivation of ovarian function through growth and development. Additionally, our study did not aim to evaluate the cystic kidney disease phenotype in detail, so further studies are needed to determine the mechanistic effects of OVX in adult Cy/+ rats on renal function outcomes.

Interestingly, in our study, OVX rats had lower, but not statistically significant, percent net phosphorus absorption that may suggest that estrogen influences intestinal phosphate transporters. Intestinal tissue is not available from the present study to directly test this mechanistic hypothesis, but there is some prior experimental evidence to support this notion. Acute 17ß-estradiol injection at 2 mg/kg body weight in rats increased intestinal brush boarder membrane vesicle uptake and the mRNA and protein expression of the main known intestinal phosphate transporter, sodium phosphate cotransporter 2b^42^. However, in contrast, a study in female rats of similar age to those in our study found no change in net phosphorus absorption with injection of 5 or 40 ug/kg 17ß-estradiol for 21 days^43^. Further research on the effects of ovariectomy and estrogen on phosphorus absorption are needed. OVX also resulted in a non-significant trend towards lower whole-body phosphorus and calcium balance. In a separate study of Cy/+ and WT male rats, we found that phosphorus and calcium balance are also marginally lower in Cy/+ males with CKD compared to normal WT controls^44^. The lower phosphorus and calcium balance in the present study is likely reflective of the loss of bone mass in the OVX rats compared with Sham.

This study has several limitations. First, this study was designed as a feasibility study to determine if OVX of Cy/+ females would produce the biochemical and bone phenotypic features of CKD-MBD that are observed in male Cy/+ rats of similar age. Thus, only two groups of heterozygous Cy/+ females, with OVX or Sham control, were studied, and we relied on historical data in male Cy/+ rats for qualitative comparisons between the sexes. Additionally, because of the study aim of establishing a practical female model of CKD-MBD, our study did not include more detailed assessments of kidney function, such as direct GFR by inulin clearance, urinary protein excretion, or renal histology including cystic lesions and interstitial fibrosis. These in-depth assessments would be needed to draw more substantial conclusions on the mechanistic effects of estrogen deficiency on renal function in this rat model but are not available from the present study. A future study aimed at determining effects of estrogen on kidney function in this model would also ideally include an OVX plus exogenous estrogen treatment group. Further explorations of sex differences and effects of gonadal hormones on kidney disease progression and features of the CKD-MBD phenotype would ideally have groups of both males and females with orchidectomy or OVX, respectively, and possibly hormonal repletion treatments. However, since we observed no overt effects of OVX on the biochemical or bone phenotype in this model, such future studies might be better conducted in other CKD models, such as the 5/6^th^ nephrectomy model.

In summary, OVX did not produce alterations consistent with the CKD-MBD phenotype as observed in our previous work in Cy/+ males as we had hypothesized. Our findings suggest that the OVX Cy/+ rat is not a practical female model for studying postmenopausal CKD-MBD. Development and validation of other female rodent models of CKD-MBD are needed.

## Supplementary information

Dataset 1

## Data Availability

Individual data generated or analyzed during this study are included in the Supplementary Information file (Supplementary Data.xlsx).
